# Biomaterials with Potential Use in Bone Tissue Regeneration—Collagen/Chitosan/Silk Fibroin Scaffolds Cross-Linked by EDC/NHS

**DOI:** 10.3390/ma14051105

**Published:** 2021-02-26

**Authors:** Sylwia Grabska-Zielińska, Alina Sionkowska, Ângela Carvalho, Fernando J. Monteiro

**Affiliations:** 1 Department of Physical Chemistry and Physicochemistry of Polymers, Faculty of Chemistry, Nicolaus Copernicus University in Toruń, 87-100 Toruń, Poland; 2 Department of Chemistry of Biomaterials and Cosmetics, Faculty of Chemistry, Nicolaus Copernicus University in Toruń, 87-100 Toruń, Poland; as@chem.umk.pl; 3 i3S—Instituto de Investigação e Inovação em Saúde, Universidade do Porto, Rua Alfredo Allen, 208, 4200-180 Porto, Portugal; angela.carvalho@ineb.up.pt (Â.C.); fjmont@i3s.up.pt (F.J.M.); 4 INEB—Instituto de Engenharia Biomédica, Universidade do Porto, Rua Alfredo Allen, 208, 4200-180 Porto, Portugal; 5 FEUP—Faculdade de Engenharia, Universidade do Porto, Rua Dr. Roberto Frias, s/n, 4200-465 Porto, Portugal

**Keywords:** EDC/NHS, collagen, silk fibroin, chitosan, three-dimensional scaffolds

## Abstract

Blending of different biopolymers, e.g., collagen, chitosan, silk fibroin and cross-linking modifications of these mixtures can lead to new materials with improved physico-chemical properties, compared to single-component scaffolds. Three-dimensional scaffolds based on three-component mixtures of silk fibroin, collagen and chitosan, chemically cross-linked, were prepared and their physico-chemical and biological properties were evaluated. A mixture of EDC (N-(3-dimethylaminopropyl)-N’-ethylcarbodiimide hydrochloride) and NHS (N-hydroxysuccinimide) was used as a cross-linking agent. FTIR was used to observe the position of the peaks characteristic for collagen, chitosan and silk fibroin. The following properties depending on the scaffold structure were studied: swelling behavior, liquid uptake, moisture content, porosity, density, and mechanical parameters. Scanning Electron Microscopy imaging was performed. Additionally, the biological properties of these materials were assessed, by metabolic activity assay. The results showed that the three-component mixtures, cross-linked by EDC/NHS and prepared by lyophilization method, presented porous structures. They were characterized by a high swelling degree. The composition of scaffolds has an influence on mechanical properties. All of the studied materials were cytocompatible with MG-63 osteoblast-like cells.

## 1. Introduction

Biomaterials based on proteins, polysaccharides and synthetic polymers can be used in biomedical applications, especially in tissue engineering to fill in small bone defects [1]. Natural polymers are widely used in tissue engineering, because of their biocompatibility, biodegradability, non-toxicity and ability to degrade inside the body without releasing toxic substances [2,3]. The ecological aspect is also relevant, from the climate disaster and environmental pollution point of view. Chitosan can be obtained from food waste, silk fibroin from textile waste, and collagen is a raw material widely available in the animal world. The newest research shows that marine collagen is being used more and more in science [4,5,6,7]. It can be used in the biomedical, pharmaceutical and cosmetic industries [3,6]. However, the limitation of this type of collagen is low denaturation temperature, and researchers are constantly working to improve its properties and find the best applications.

Collagen, chitosan and silk fibroin were used to prepare single- and two-component materials such as films, fibers, three-dimensional scaffolds and hydrogels [8,9,10,11,12]. Nevertheless, materials made of a single component have several disadvantages. They have poor stability in water and physiological conditions and low mechanical parameters [2]. Therefore, there is a constant need to work on better materials. To improve the specific parameters useful in tissue engineering, two or more polymers can be mixed together to obtain new materials. Many two-component mixtures have already been investigated: chitosan/collagen [9], chitosan/gelatin [13], chitosan/hyaluronic acid [14], alginate/collagen [15]. Additionally, three-component mixtures were prepared and analyzed for potential biomedical applications: collagen/chitosan/hyaluronic acid [16], alginate/chitosan/collagen [17] or carrageenan/chitosan/gelatin [18].

However, an even better way to improve the physico-chemical parameters of biopolymeric materials is cross-linking. The main aim of the cross-linking process is to overcome the limitations of biomaterials by interconnecting molecules, increasing molecular weight and generation of intermolecular interactions [19,20]. Cross-linking is the most common approach to modifying polymeric materials. Three types of cross-linking can be classified: enzymatic, physical and chemical cross-linking [2,19].

Modification and cross-linking of polymers and biopolymers, using enzymes has captured high interest among research groups [21]. Reactions with enzymes are characterized by high specificity, lack side products and low energy demand. Transglutaminase, laccase, tyrosinase and horseradish peroxidase can be used for enzymatic cross-linking [19,22].

A physical cross-linking process usually leads to the change of physicochemical properties of the polymer through the action of physical factors. This kind of cross-linking includes different techniques, e.g., UV irradiation, Gamma radiation, laser treatment, de-hydrothermal treatment [2,19,22]. Physical modification is a simpler and cheaper method compared to other processes of polymer modification [23].

Chemical cross-linking is the process used to covalently bridge polymeric chains to improve polymer properties [19,24]. There are a lot of cross-linking agents used for chemical cross-linking, e.g., glutaraldehyde, genipin, dialdehyde starch, glyoxal, EDC/NHS (N-(3-dimethylaminopropyl)-N’-ethylcarbodiimide hydrochloride/N-hydroxysuccinimide), [25,26,27,28,29,30]. However, unfortunately, there is a risk of toxic effect by chemical cross-linkers [31].

This problem, allied with ecological challenges, prompted us to develop new materials for use in tissue engineering. We decided to create new scaffolds based on collagen, chitosan and silk fibroin, cross-linked by an EDC/NHS mixture to determine several physico-chemical properties and cellular response to this type of material. The basic research on the physico-chemical properties measurements and in vitro study on MG-63 osteoblast-like cells of materials were prepared. In an aim to check if the materials designed in this way—based on three-component blends (silk fibroin, collagen, chitosan) cross-linked by EDC/NHS mixture, would be suitable for bone tissue engineering. In vitro test with using MG-63 cells was performed in an aim to see if the materials do not toxic effect on cells and to check if there is unreacted residual of cross-linker in the material that could be toxic to cells and in the future application would give rise to an immune response in the human body. However, it should be emphasized that in vitro study needs to be followed by tests on animals before human clinical trials.

**Figure 1:**
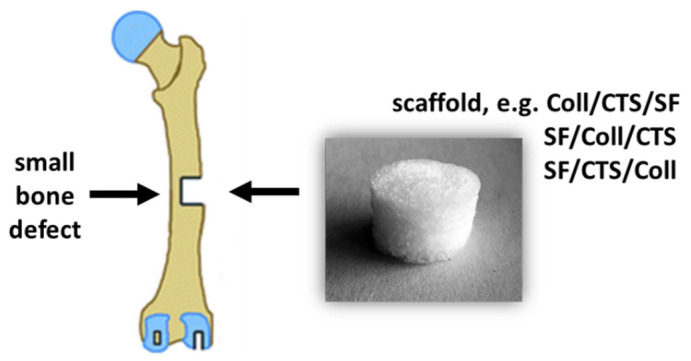
The graphical presentation of the possible use of the material.

**Figure 2:**
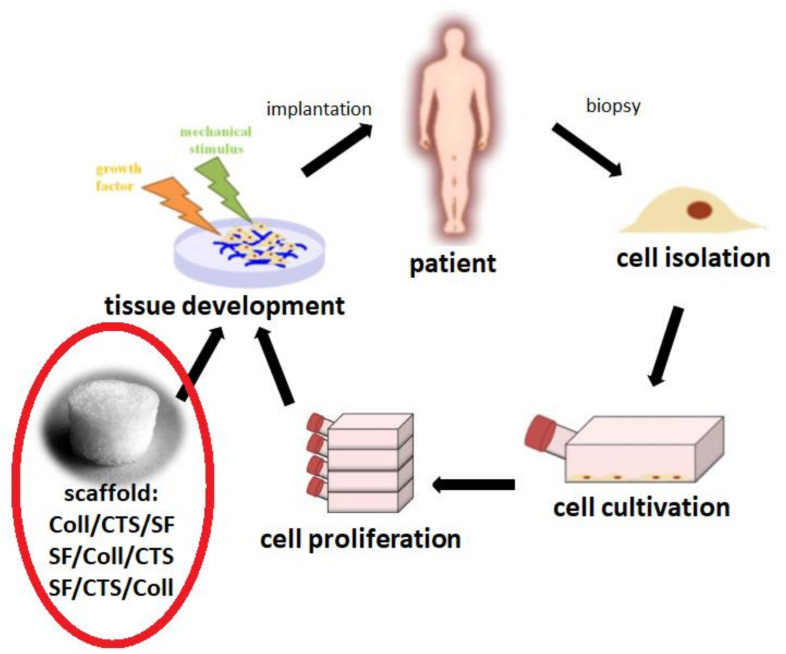
The steps of the tissue engineering process. In the red circle, the part of the tissue engineering round to which the article relates is crossed—design, physico-chemical characterization and in vitro study on cells of materials consisting of ternary mixtures based on silk fibroin, collagen and chitosan cross-linked with EDC/NHS mixture.

Silk fibroin, collagen and chitosan materials in various compositions and weight ratios could be used in bone tissue engineering to fill small bone defects resulting from diseases, traumas, injuries, infections and genetic defects (Figure 1) [32,33]. Treatment of bone defects are a big challenge for modern medicine. The biggest thing of research in biomaterials for tissue engineering is to design and prepare suitable three dimensional scaffold and finding a new, adequate solutions is extremely important (Figure 2) [34]. The latest trend in biomedical engineering is the search for mixtures based on natural polymers that would demonstrate compatibility with human tissues [35]. Natural polymers are used for the production of three-dimensional tissue scaffolds, due to their degradability to non-toxic components, biocompatibility, bioactivity and bioresorbability.

Scaffolds based on silk fibroin and silk fibroin with 0.05% and 0.01% addition of collagen with a surfactant as a substance to gelation were studied by Apinun et al. [36]. Collagen combined in the hydrogels yielded a positive effect on matrix formation and proliferation. However, the authors said that there have to be more studies in order to better understand how to optimize the system as a three-dimensional material and cell carrier in bone tissue regeneration [36]. On the other hand, according to Zeng et al. [37], materials composed form silk fibroin and collagen in 40/60 and in 60/40 weight ratio were good biomaterials for bone tissue engineering. In this report, the in vitro cell proliferation using MG-63 cells has been studied. The pore sizes and porosity (above 90%) were adequate for the growth of osteoblasts and the rate of degradation was steady [37]. Following this work, where silk fibroin and chitosan scaffolds were studied with 3T3 fibroblast cells, 3D materials were biocompatible [38]. Taking into account the different compositions of materials, the best mechanical properties were observed in SF/CTS 20/80 blend scaffold [38]. The SF/CTS 50/50 scaffold was selected to test the inflammatory response in vivo and it was no obvious inflammatory response. The study was performed after two and four weeks, and it was noticed that components and structure of the SF/CTS material were beneficial for the formation of new blood vessels, cell adherence and in-growth [38].

## 2. Materials and Methods

### 2.1. Materials

Sodium carbonate (Na_2_CO_3_), lithium bromide (LiBr), acetic acid (CH_3_COOH), chitosan (DD = 78%; viscosity average molecular weight = 0.59 × 106 g/mol), (N-(3-dimethylaminopropyl)-N’-ethylcarbodiimide hydrochloride, N-hydroxysuccinimide, calcium chloride dihydrate (CaCl_2_ × 2H_2_O), phosphate buffered saline (purchased in a tablet form with one tablet dissolved in 200 mL of deionized water yields 0.01 M phosphate buffer, 0.0027 M potassium chloride and 0.137 M sodium chloride, pH 7.4, at 25 °C), ethanol (C_2_H_5_OH) and di-sodium phosphate (Na_2_HPO_4_) were purchased from Sigma-Aldrich (St. Louis, MO, USA). 

### 2.2. Preparation of Silk Fibroin

Silk fibroin was prepared from *Bombyx mori* cocoons (Jedwab Polski Sp. z o.o., Milanówek, Poland). The procedure was the same as in Quiang et al. [39], with small modifications. First, the cocoons were boiled in a Na_2_CO_3_ (C = 0.5%) aqueous solution for 1 h. This step was performed twice. Then, the cocoons were boiled in an alkaline soap solution (C = 5%) for 30 min. Next, the silk was boiled in distilled water for 20 min and rinsed thoroughly with distilled and tap water to extract the sericin proteins. That whole procedure was repeated three times. Subsequently, the degummed silk was dried at room temperature and humidity for 48 h.

### 2.3. Preparation of Collagen

Collagen was isolated from tail tendons of young rats, following A. Sionkowska and J. Kozłowska method [40]. The rat tails were the waste from other investigations on rats, which were performed with the agreement of Bioethical Commission. The tendons were separated from adhering tissues, washed in distilled water twice and dissolved in aqueous acetic acid solution (C = 0.1 M) for 3 days at 4 °C. The insoluble parts were separated by centrifugation (10,000 rpm, Eppendorf AG, Hamburg, Germany). Collagen solution was freeze-dried to obtain 100% pure collagen (ALPHA 1–2 LDplus, CHRIST, −20 °C, 100 Pa, 48 h).

### 2.4. Preparation of Three-Component Scaffolds

Collagen, chitosan and silk fibroin were used to obtain three-dimensional scaffolds. Silk fibroin was dissolved in lithium bromide (C = 9.3 M) for 4 h at 80 °C and filtered. The final silk fibroin concentration was 5%. The collagen lyophilizate was dissolved in 0.1 M acetic acid aqueous solution to 1% final concentration. Chitosan was prepared as 1% concentrated solution in 0.1 M acetic acid aqueous solution. Two of the three biopolymers were mixed together in 50/50 weight ratio with a magnetic stirrer for 2 h. The third biopolymer was added in 10, 20 and 30 *w*/*w*% addition and mixed for 3 h. Afterwards, the mixtures were dialyzed against distilled water for 3 days to aqueous solutions. The dialyzed solutions were poured to 24-well polystyrene cell culture plates and frozen overnight in a −80 °C freezer, followed by lyophilization (−55 °C, 5 Pa for 48 h, ALPHA 1–2 LD plus, CHRIST, Germany). Three types of mixtures were prepared. Each type of mixtures was based on 50/50 weight ratio mixture: Coll/CTS; SF/Coll; SF/CTS. Third biopolymer was added to mixture with 10, 20 and 30% amount. Each scaffold was cross-linked by EDC/NHS.

### 2.5. Cross-Linking of Scaffolds

**Figure 3:**
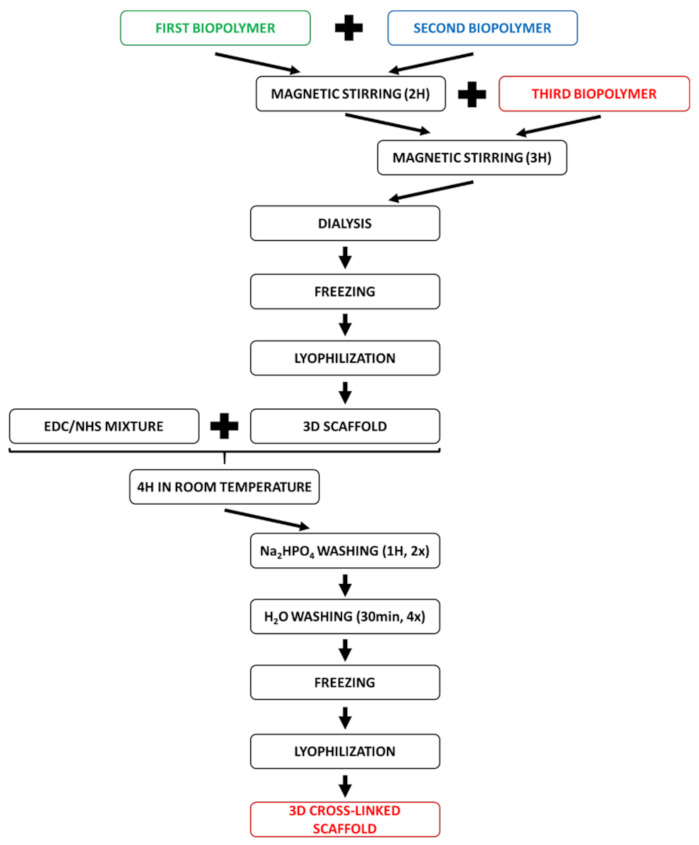
The scheme of preparing and cross-linking biopolymeric scaffolds.

The mixture of EDC and NHS (N-(3-dimethylaminopropyl)-N’-ethylcarbodiimide hydrochloride and N-hydroxysuccinimide) was used as cross-linking agent. The mixture of EDC and NHS (50 mM EDC and 25 mM NHS) in 98% ethanol was prepared. The obtained scaffolds were poured in cross-linking mixture for 4 h in room temperature. After the reaction, the scaffolds were washed by 0.1 M Na_2_HPO_4_ for 1 h, twice. Then, the scaffolds were washed with deionized water for 30 min, four times [41]. After washing, the scaffolds were frozen and lyophilized in the same way as previously described. The scheme of preparing and cross-linking scaffolds is shown in Figure 3.

### 2.6. Chemical Structure Studies—Fourier Transform Infrared Spectroscopy (FTIR)

The chemical structure of the obtained scaffolds was studied using a Nicolet iS10 spectrophotometer equipped with an ATR device with a germanium crystal (Thermo Fisher Scientific, Waltham, MA, USA). All the spectra were recorded with a resolution of 4 cm^−1^ with 64 scans. The spectra were evaluated in the range of 650–4000 cm^−1^. The data were collected and plotted using the Omnic program.

### 2.7. Swelling Properties, Liquid Uptake and Moisture Content

Swelling degree was measured by immersing the scaffolds fragments in 10 mL of phosphate-buffer saline (PBS) solution, pH = 7.4 at 37 °C (incubator, Sanyo, Tokyo, Japan). After 1, 2, 24, 72, 168 h of immersion, materials were softly dried (by putting them between two sheets of paper) and weighted [42]. The swelling degree was calculated using the following equation:

$$swelling\;\left[\%\right]=\frac{m_{s\left(t\right)}-m_{s\left(0\right)}}{m_{s\left(0\right)}}\times 100\%$$

*m_s_*_(*t*)_—the weight of the material after immersion in PBS;

*m_s_*_(0)_—the weight of the material before immersion.

Liquid uptake is the percentage of liquid content after the scaffolds’ immersion. It is related with the scaffolds’ weight changes. The scaffolds were placed in PBS (phosphate-buffer saline mass (*m*_0_)) and then, after 1, 2, 24, 72 and 168 h, they were removed without squeezing. The rest of the PBS solution was weighed (*m_t_*) and the liquid uptake was calculated [43]:

$$liquid\;uptake\;\left[\%\right]=\left|\frac{m_{t}-m_{0}}{m_{o}}\right|\times 100\%$$

Scaffold moisture content was measured by drying samples in an oven (DZ-2BC Vacuum Oven, ChemLand, Stargard, Poland) at 105 °C until they reached a constant weight. The weighing results were expressed as grams of water per 100 g of dry sample [44]. Samples of each type were measured in triplicate.

### 2.8. Microstructure Studies—Scanning Electron Microscopy, Porosity, Density

To observe microstructure of scaffolds, Scanning Electron Microscopy was used. Images were taken by Scanning Electron Microscope (LEO Electron Microscopy Ltd., England, UK). The fragments of samples were frozen for 3 min in liquid nitrogen. This kind of freezing allows mild cutting with a razor scalpel. The prepared samples were covered with gold and SEM images were made with 200 and 500 μm resolution. The microstructure of the samples after and before the swelling process in PBS were studied and compared.

Porosity and density measurements can also be used to assess the microstructure of scaffolds. To measure these parameters, liquid displacement method was used. Isopropanol was used as the liquid. A fragment of a porous material with a known weight (W) was immersed in a cylinder with a known volume of isopropanol (*V*_1_) for 3 min. The volume of isopropanol with the material was measured (*V*_2_) and the volume of isopropanol after the removal of the sample (*V*_3_) was measured, too. The porosity was calculated using the following equation:

$$\varepsilon \;\left[\%\right]=\;\frac{V_{1}-V_{3}}{V_{2}-V_{3}}\times 100\%$$

The density was calculated using the following equation:

$$d\;\left[\frac{mg}{cm^{3}}\right]=\;\frac{W}{V_{2}-V_{3}}$$

*W*—sample weight [mg],

*V*_1_—initial volume of liquid [cm^3^],

*V*_2_—total volume of liquid and liquid impregnated sample [cm^3^],

*V*_3_—isopropanol volume after scaffold removal [cm^3^].

### 2.9. Mechanical Properties Studies

Zwick&Roell 0.5 testing machine (Zwick&Roell Group, Ulm, Germany) was used to measure the mechanical properties of biopolymer scaffolds. Mechanical measurements were performed in room conditions (temperature, humidity). Five samples of each kind of scaffolds were studied. Mechanical testing was doing with crosshead speed set at 0.5 mm/min. For each kind of material, 5 samples were measured and the standard deviation was calculated. The scaffold was placed between two discs of the testing machine and compressed. The diameter of the material was 14.44 mm with the thickness in the range of 13.0–15 mm.

### 2.10. Biological Studies—In Vitro Cytocompatibility Assay—Metabolic Activity

The cytocompatibility of the scaffolds was evaluated on MG-63 osteoblast-like cells’ metabolic activity. The MG-63 (ATCC^®^ CRL-1427™) cell line was provided by ATCC. ATCC full name is American Type Culture Collection (Manassas, VA, USA). The cells were cultured in α-MEM (alpha modification of Eagle minimum essential medium, Sigma-Aldrich) supplemented with 10% fetal bovine serum (FBS, Gibco), 1% penicillin-streptomycin (3 × 10^−4^ mol/L and 5 × 10^−4^ mol/L, Gibco) and maintained in a humidified atmosphere at 37 °C and 5% of carbon dioxide (CO_2_), with the culture medium changed twice a week. When a high degree of confluence was reached, the adherent MG-63 cells were washed with PBS, enzymatically released with 0.04% trypsin and subcultured. Thin fragments of scaffolds (about 14 mm in diameter and 3 mm in height) were sterilized by soaking with a 70% solution of ethanol and washed three times with sterile PBS (pH = 7.4, Sigma Aldrich).

The scaffolds were seeded with 1 × 10^4^ cells per sample (suspended in 1 mL of media) in 24-well cell culture plates. As a control, the cells were seeded directly on TCPS. The viability of the cells was determined using resazurin reduction assay. The measurements were made at 1, 4 and 7 days of culture. A total of 10% (*v*/*v*) concentrated resazurin was added to the medium and incubated for 4 h at 37 °C and 5% of CO_2_. After this time, 100 µL mixture of media with resazurin was transferred into a black 96-well plate, and the fluorescence was measured at 530 nm excitation and 590 nm emission wavelength. A fluorescence reader (SynergyMix, BioTek, Winooski, VT, USA) with Gen5 1.09 Data Analysis Software was used. The results are expressed as relative fluorescent units (RFU). Scaffolds of each type were measured in triplicate.

### 2.11. Statistical Analysis

Statistical analysis of the data was completed using commercial software GraphPad Prism 8.0.1.244, GraphPad Software, San Diego, CA, USA. The results were presented as a mean ± standard deviation (SD) and were statistically analyzed using one-way analysis of variance (one-way ANOVA). Multiple comparisons between the means were performed with the statistical significance set at *p* ≤ 0.05.

## 3. Results and Discussion

### 3.1. Chemical Structure Studies—Fourier Transform Infrared Spectroscopy (FTIR)

**Figure 4:**
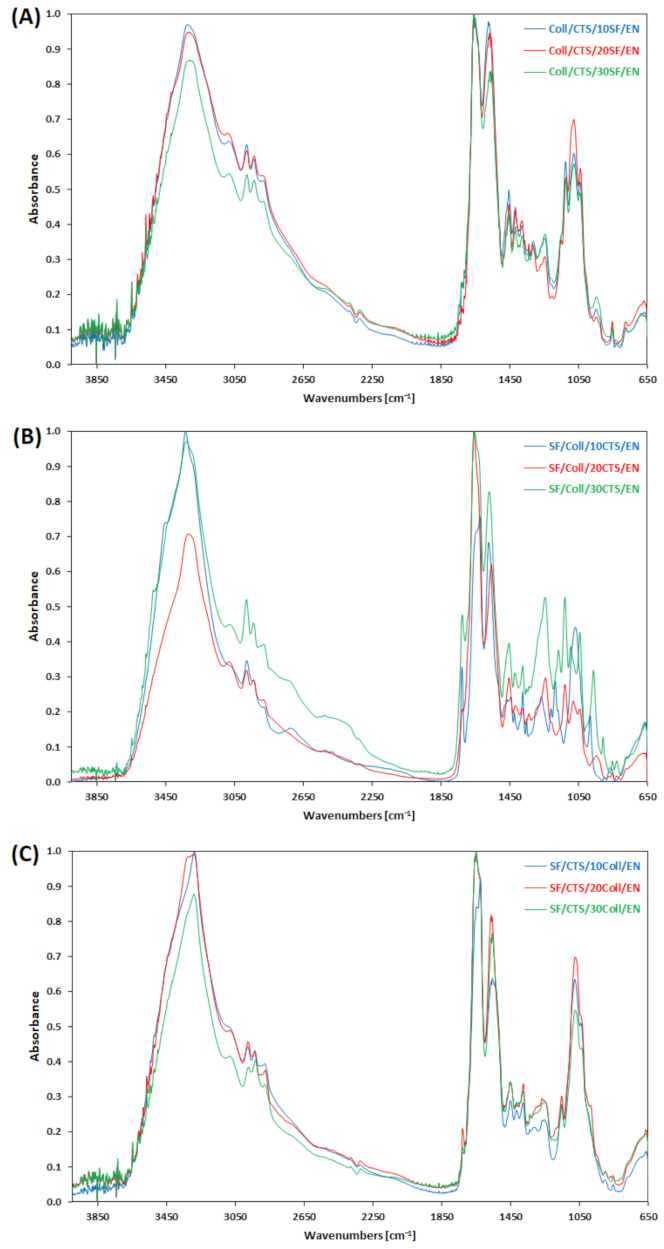
FTIR-ATR spectra of biopolymeric scaffolds: (**A**) 50/50 Coll/CTS mixtures with 10, 20, and 30% of SF; (**B**) 50/50 SF/Coll mixtures with 10, 20, and 30% of CTS; (**C**) 50/50 SF/CTS mixtures with 10, 20, and 30% of Coll.

Fourier transform infrared spectroscopy can be a useful technique for studying the natural polymers structure [41]. The FTIR-ATR spectra, recorded for each of the tested materials, are presented in Figure 4. Collagen, chitosan and silk fibroin belong to the biopolymer group and for this reason in FTIR spectra these biopolymers show similar bands [45]. Some specific bands of collagen, chitosan, silk fibroin-based mixtures can be observed. The characteristic bands: amide I, II and III were noticed around 1650 cm^−1^, 1537 cm^−1^ and 1241 cm^−1^, respectively [45]. The Amide A band, around 3285 cm^−1^, was presented in FTIR spectra of each material. The characteristic band for collagen, Amide B, after the cross-linking process could not be precisely located. It is the proof that the bond between polymers and cross-linker includes this group [41]. At ~1050 cm^−1^ C-O-C stretching vibrations peak, which is responsible for pyranose ring, was observed [46]. Materials based on collagen, chitosan and silk fibroin, cross-linked by EDC/NHS, show similar positions of characteristic peaks in FTIR spectra. There are no significant differences in band locations. Only some intensity changes of the bands can be observed.

### 3.2. Swelling Properties, Liquid Uptake and Moisture Content

**Figure 5:**
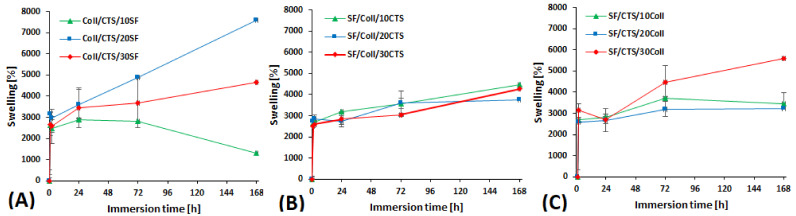
Swelling behavior of the studied scaffolds, cross-linked by EDC/NHS: (**A**) Coll/CTS mixture with 10, 20 and 30% of SF; (**B**) SF/Coll mixture with 10, 20 and 30% of CTS; (**C**) SF/CTS with 10, 20 and 30% of Coll.

Hydrophilic and porous materials are characterized by a high swelling degree [47]. Swelling properties measurements mimic the behavior of the material after its application into the human body. PBS (phosphate-buffer saline) was the liquid used to the measure swelling ratio (pH = 7.4, relevant to the pH of blood) [40]. The materials made of collagen, chitosan and silk fibroin are easily wettable by polar solvents such as PBS [44]. The swelling ratio results for the studied materials are shown in Figure 5. The measurements were carried out at several timepoints: 1 h, 2 h, 24 h, 72 h and 168 h. The materials made of collagen, chitosan and silk fibroin contain a lot of functional groups capable of binding water, and that is why they exhibit a high swelling ability. For each material, the swelling ratio increased considerably during the first two hours. A big increase in swelling behavior, in the range of 700 to 1000%, after 1 h was reported by Panjapheree et al. [48]. They studied SF, SF70:30CTS, SF50:50CTS, SF30:70CTS and CTS scaffolds. Scaffolds prepared from mixtures of silk fibroin and chitosan were characterized by higher swelling ability than silk fibroin-based scaffolds [48]. After 6, 12 and 24 h, stabilization of swelling ratio was observed for materials made of SF70:30CTS and pure silk fibroin. Swelling ratio for rest mixtures was also stabilized, but it was higher after 12 and 24 h than after 6 h [48]. For materials made of SF/Coll (50/50), with the addition of chitosan, it stabilizes at approximately 3500%. This observation is in good agreement with other research on binary and ternary blends [49,50]. A different situation was observed for the materials made of collagen/chitosan (50/50) with addition of silk fibroin and for the materials made of silk fibroin/chitosan (50/50) with addition of collagen. In those cases, the swelling ratio was strictly dependent on materials composition. Especially for Coll/CTS/10SF material, the swelling became smaller with increasing of immersing time. This is because of the degradation. The degradation process is going, the scaffold decomposes and its smaller surface area is capable to absorb PBS. Even with these kinds of results, the swelling ratio is high, and one may consider that the swelling ratio for three-component mixtures is higher than for materials made of one or two biopolymers [51,52]. Lin et al. [53] reported that swelling ratio is dependent on silk fibroin and collagen content in membrane materials. It was noticed that swelling percentage of the composite decreases as the content of collagen reduces. The SF50:50Coll, SF70:30Coll and SF90:10Coll compositions of the materials were studied. The highest swelling ratio was reported for SF50:50Coll membrane [53]. Our research for materials based on 50/50 SF/CTS mixture with various content of collagen is consistent with this conclusion. The swelling ratio is higher for SF/CTS/30Coll than SF/CTS/10Coll and SF/CTS/20Coll. It can be a result of hydrophilicity of collagen which can maintain a high amount of water. The highest swelling ratio was observed for Coll/CTS/20SF (7580 ± 50%). The lowest swelling ratio, after 168 h of immersion, was observed for Coll/CTS/10SF (1302 ± 30%) and was lower than the swelling ratio after 24 and 72 h of immersion.

**Figure 6:**
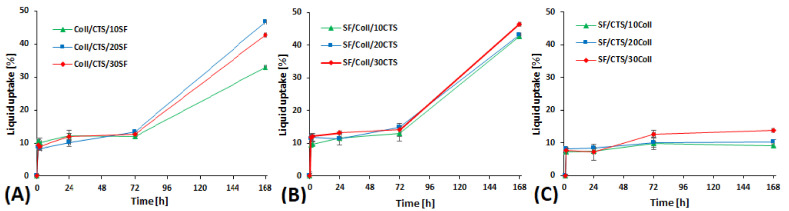
Result of liquid uptake in the studied scaffolds, cross-linked by EDC/NHS: (**A**) Coll/CTS mixture with 10, 20 and 30% of SF; (**B**) SF/Coll mixture with 10, 20 and 30% of CTS; (**C**) SF/CTS with 10, 20 and 30% of Coll.

The results of the liquid uptake are shown in Figure 6. The liquid uptake is strictly related with scaffold porosity. A higher degree of liquid uptake may suggest that the material presents higher porosity [54]. Liquid uptake is dependent on the composition of the material. Each material, after 2 h, absorbed around 10% of PBS in which they were immersed. For mixtures made of collagen/chitosan with addition of silk fibroin and made of silk fibroin/collagen with addition of chitosan, after 24 and 72 h the liquid uptake was stable and was in the range of 11.5 to 15%. Between measurements on the 3rd and the 7th day, the liquid absorption of these materials increased significantly. In the case of silk fibroin-/chitosan-based materials, liquid uptake was at the same level after 2, 24, 72 and 168 h. The scaffold degradation process caused by PBS salt had already been initiated, which is observed as liquid uptake stabilization or decrease [55].

**Figure 7:**
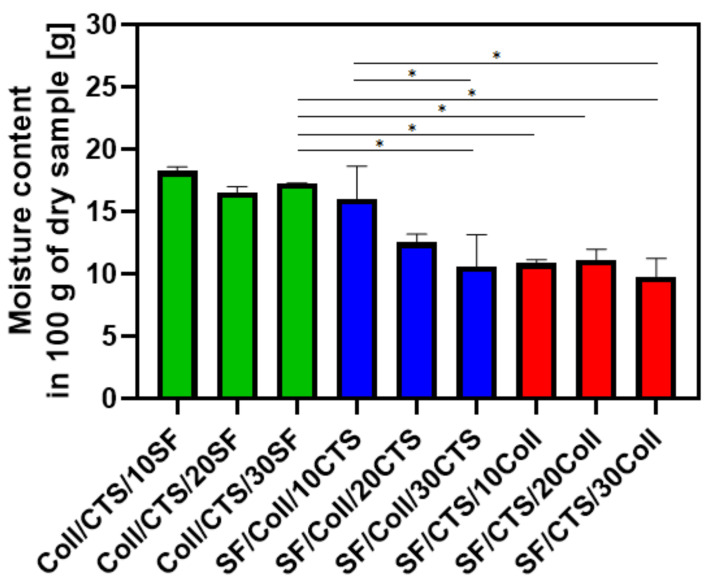
The result of moisture content measurements of biopolymeric three-component scaffolds cross-linked by EDC/NHS; *—represents statistically significant differences between samples (*p* ≤ 0.05).

The results of the moisture content of the scaffolds are shown in Figure 7. The results are presented as grams of moisture per 100 g of dry sample. Materials based on collagen/chitosan (50/50) mixture with addition of silk fibroin, are characterized by the highest moisture content. For mixtures of silk fibroin/collagen, as the content of chitosan increases, the water content decreases. Moisture content for mixtures based on silk fibroin/chitosan, are characterized by a similar ratio, around 10 g/100 g. Moisture content higher than 10 g/100 g is typical of materials based on biopolymers, cross-linked, for example, by dialdehyde starch or glyoxal [44,52].

### 3.3. Microstructure Studies—Scanning Electron Microscopy, Porosity, Density

**Figure 8:**
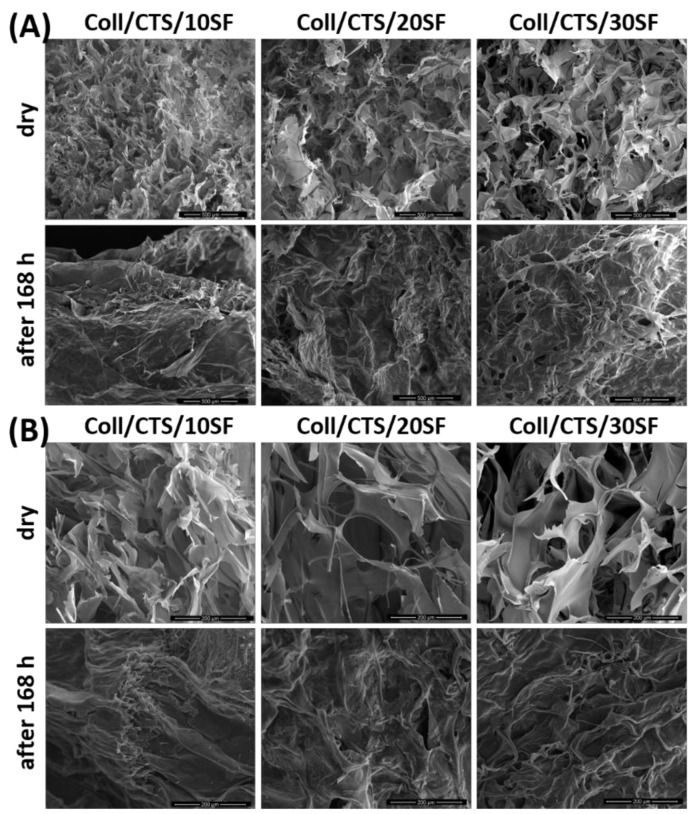
SEM images of Coll/CTS mixtures with 10, 20 and 30% of SF, cross-linked by EDC/NHS, for dry scaffolds and scaffolds after 168 h of immersion in PBS: (**A**) magnification 500×; (**B**) magnification 150×.

**Figure 9:**
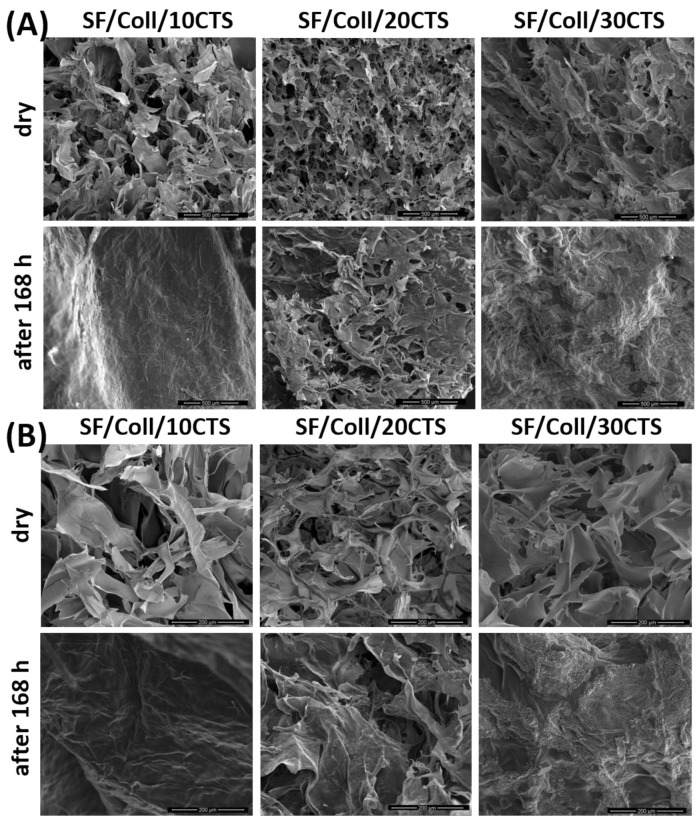
SEM images of SF/Coll mixtures with 10, 20 and 30% of CTS, cross-linked by EDC/NHS, for dry scaffolds and scaffolds after 168 h of immersion in PBS: (**A**) magnification 500×; (**B**) magnification 150×.

**Figure 10:**
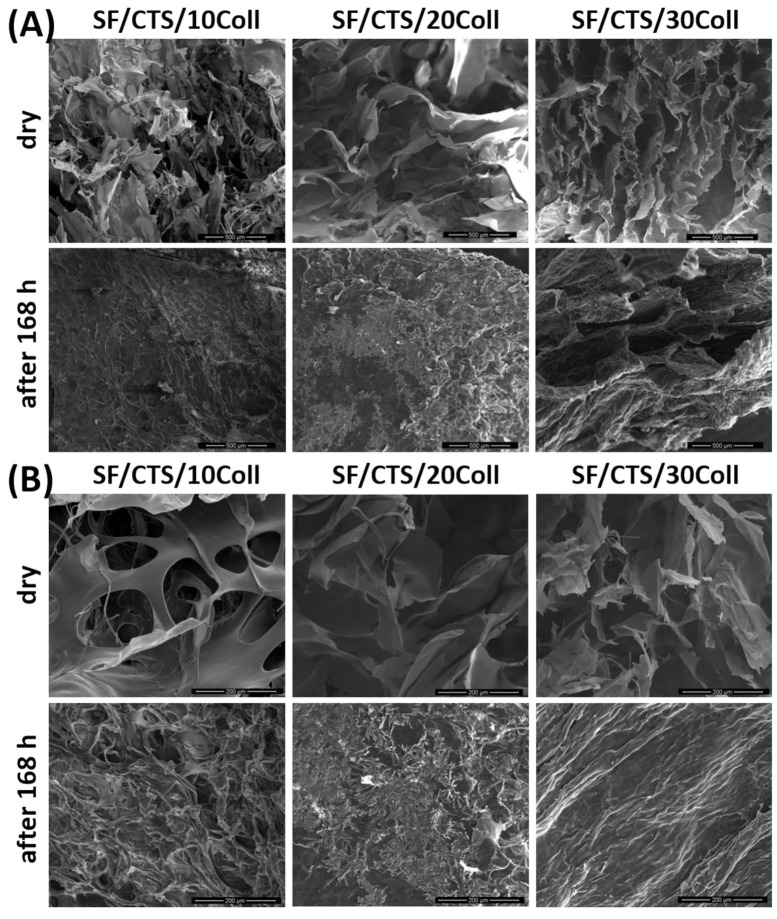
SEM images of SF/CTS mixtures with 10, 20 and 30% of Coll, cross-linked by EDC/NHS, for dry scaffolds and scaffolds after 168 h of immersion in PBS: (**A**) magnification 500×; (**B**) magnification 150×.

The microstructure of the biopolymeric materials, before and after 168 h of PBS immersion was evaluated by Scanning Electron Microscopy (Figure 8, Figure 9 and Figure 10). Scaffolds after immersion in PBS were dried at room temperature and humidity. Magnifications of 500× and 150× were used in this study. It can be observed that each kind of dry material has a porous structure with interconnected pores. These looked similar to other porous structure [56,57,58] scaffolds. This kind of structure is required for biomedical applications with three-dimensional scaffolds [59]. The suitable microstructure and pore size, provide migration cells, transport nutrients and metabolites, signaling molecules and cellular waste [58]. Thanks to SEM imaging, pore size can be calculated approximately [60,61]. The diameters of pores are equal or less than 200 μm. As can be found in the literature, the required minimum pore size for biomedical applications is about ~100 μm [62]. However, a bigger number of blood vessels was observed to grow when the pore diameter was larger than 100–200 μm [60]. According to our previous reports, the pores had similar sizes to those found in collagen/hyaluronic acid/chitosan-based materials [63] or materials based on chitosan/collagen/silk fibroin cross-linked by glyoxal [52] and dialdehyde starch [44]. The pore size was also in similar ranges, e.g., made of silk fibroin/collagen/hyaluronic acid (pore size approximately 100–300 µm) [52], silk fibroin microfibers/poly(glycerol sebacate), chitosan/poly(glycerol sebacate) (pore size approximately 50–250 µm) [53] or silk fibroin/chitosan/gelatin (pore size approximately 70–280 µm) [54] scaffolds. However, there were no significant changes between biopolymeric scaffolds based on silk fibroin, collagen and chitosan. Scanning Electron Microscopy imaging was also used to observe the degradation process [52,55]. After 168 h of PBS immersion, the microstructures of the materials were completely different. It was irregular, the pores were not so clear, large, opened, and the edges of the pores were more rounded. It can be seen that the degradation process resulting from the presence of PBS salts was initiated during the 168 h immersion (Figure 8, Figure 9 and Figure 10) [54]. In addition, for some scaffolds (Figure 8—Coll/CTS/20SF; Figure 9—SF/Coll/20CTS; SF/CTS/20Coll), salt precipitation could be observed. Potassium, sodium and phosphate ions are present in the PBS solution. This is a rare and atypical process, but it may provide the materials with high biocompatibility.

**Figure 11:**
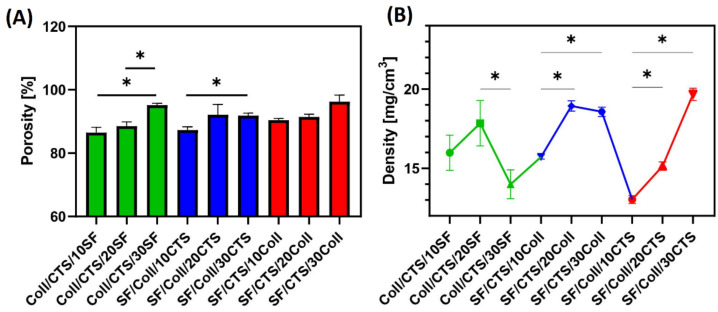
Results of (**A**) porosity and (**B**) density measurements of biopolymeric three-component scaffolds cross-linked by EDC/NHS; *—represents statistically significant differences between samples (*p* ≤ 0.05).

The porosity and density were measured for three-component materials, cross-linked by EDC/NHS, and their results are presented in Figure 11. It is known that a porosity of about 80–90% is the appropriate value for materials to be used in bone tissue regeneration applications [64]. It is the best microstructure for scaffolds, because high porosity can provide sufficient nutrient and gas exchange and enough space for cell proliferation and attachment [64]. The highest porosity was observed for SF/CTS/30Coll scaffold (just over 90%). Similar values of porosity were observed for Coll/CTS/30SF material. Similar levels of porosity were present in scaffolds made of silk fibroin and mixture of silk fibroin/chitosan/gelatin, where their porosities were 92% and 83%, respectively [58]. With respect to silk fibroin microfibers/poly(glycerol sebacate) and chitosan/poly(glycerol sebacate) scaffolds, porosity was around 42% [57]. It is a completely different situation for materials made of silk fibroin and chitosan, according to Zeng et al., where the porosity achieved was about ~95% [37]. According to the report by Li et al. [65], when silk fibroin, silk fibroin/chitosan, silk fibroin/gelatin and silk fibroin/chitosan/gelatin scaffolds were prepared, it could be seen that porosity was dependent on the composition of the scaffold. They observed the highest porosity in silk fibroin/chitosan materials (77.48 ± 1.27%) and the lowest in silk fibroin/chitosan/gelatin materials (41.67 ± 8.33%), whereas silk fibroin/chitosan/gelatin scaffolds and one-component silk scaffolds were characterized with 42.06 ± 4.83% and 60.74 ± 3.23% porosity, respectively [65]. All ternary materials, cross-linked by EDC/NHS, were characterized by porosity higher than 80%. For materials based on Coll/CTS with addition of silk fibroin and materials based on SF/CTS with an addition of collagen, porosity increased with increasing content of the third component.

The highest density of the scaffolds was observed for SF/Coll/30CTS (19.69 ± 0.28 mg/cm^3^). The group of scaffolds based on 50/50 silk fibroin/collagen mixture with addition of chitosan was characterized by density dependence, i.e., as the addition of the third component increased, so did the density. Increasing the density of the materials could favor proliferation and cell growth, as more materials may become accessible to the cells [66].

### 3.4. Mechanical Properties Studies

**Figure 12:**
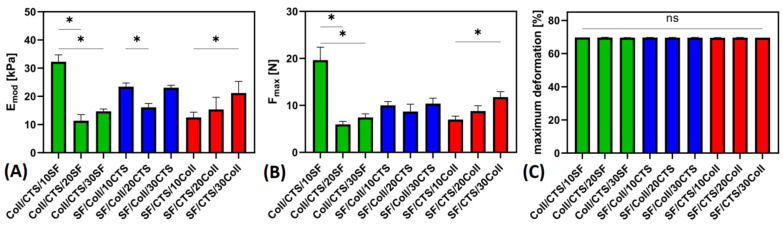
The results of mechanical properties studies of biopolymeric scaffolds, cross-linked by EDC/NHS: (**A**) Young’s modulus; (**B**) maximum force; (**C**) maximum deformation; *—represents statistically significant differences between samples (*p* ≤ 0.05); ns—represents no statistically significant differences (*p* ≤ 0.05).

Mechanical properties studies were carried out under room temperature and humidity conditions. Young’s modulus (E_mod_), maximum force (F_max_) and maximum deformation were determined. The results of mechanical parameters are shown in Figure 12. The Young’s modulus is the most frequently assessed and compared mechanical parameter for three-dimensional materials. It gives information about the flexibility and hardness of the samples [42]. The composite material mainly plays the role of support when it is used as a scaffold. It is only the transition stage material [53]. The highest Young’s modulus was observed for Coll/CTS/10SF scaffold. Completely opposite behavior was found for the same materials, when cross-linked with glyoxal, according to our previous report [52]. The poorest Young’s modulus was obtained for Coll/CTS/20SF sample. Both for the materials based on collagen/chitosan (50/50) and those based on silk fibroin/collagen (50/50) adding 20% of third biopolymers produced the lowest Young’s modulus of the respective type of materials. For scaffolds based on silk fibroin/chitosan (50/50) mixture, Young’s modulus increased with increasing addition of the third biopolymer. The same relationship was observed for the maximum force. The highest maximum force was observed for Coll/CTS/10SF and the lowest for Coll/CTS/20SF. For this parameter, too, in the case of materials based on collagen/chitosan (50/50) and based on silk fibroin/collagen (50/50), 20% additions of a third biopolymer produced the lowest maximum force of the respective type of materials. The maximum force increases with increasing addition of the third biopolymer for silk fibroin/chitosan-based scaffolds. Maximum deformation is a parameter showing how much of the material was deformed when maximum force was applied, and did not present significant differences, compared to three-component materials cross-linked by glyoxal [52]. Asadpour et al. [58] studied the mechanical properties of silk fibroin/chitosan/gelatin scaffolds in wet conditions, and they noticed that in composite scaffolds, the tensile strength (from 7/4 to 18 KPa) and elastic modulus (from 27 to 60 KPa) gradually increased when the silk fibroin content increased. The excellent mechanical strength of silk fibroin let to improvement of mechanical parameters on the composite scaffolds, which is evident in our research comparing them to pure collagen materials [67]. In Lin et al. [53] the mechanical properties of silk fibroin-collagen type II composite membrane were studied. In this report, it was found that the stress-strain curves were not coincident, which means that there are many differences with respect to the mechanical properties of composite membranes with different ratios. This is another proof that silk fibroin has the effect of improving the mechanical properties of the material—the Young’s modulus of composite membranes increases with decrease of the collagen and the nearly 2.5 times difference between the SF50 and SF90 was observed [53]. On the other hand, according to Panjapheree et al. [48], silk fibroin and SF70:30CTS scaffolds had more stress at maximum load than that of SF50:50CTS, SF30:70CTS, and CTS. They reported also that the stress at maximum load decreased as the percentage of chitosan increased. Young’s modulus of silk fibroin/chitosan materials decreased when the amount of chitosan increased and for SF50:50CTS and SF30:70CTS it was lower than the others [48]. Sun et al. [68] compared the mechanical properties of silk fibroin/collagen and silk fibroin/chitosan materials, and they reported that Young’s modulus of silk fibroin/collagen was significant higher than of silk fibroin/chitosan (*p* < 0.05). Thus, it has been suggested that silk fibroin/collagen scaffolds exhibit a higher comprehensive property than silk fibroin/chitosan and are suitable for cell cultivation [68,69].

### 3.5. Biological Studies—In Vitro Cytocompatibility Assay—Metabolic Activity

**Figure 13:**
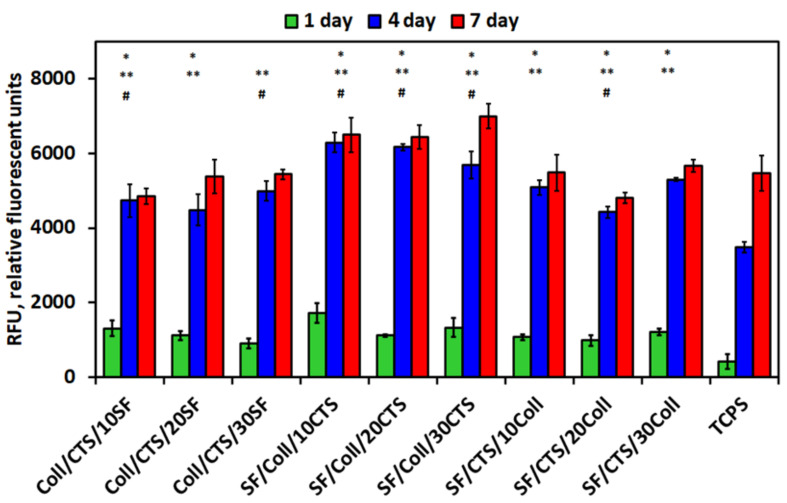
Metabolic activity after 1, 4 and 7 days of MG-63 cells cultured on studied scaffolds (n = 3, mean ± SD; *p* ≤ 0.05; *—significantly different between sample and TCPS in 1 day; **—significantly different between sample and TCPS in 4 days; #—significantly different between sample and TCPS in 7 days).

Three-dimensional artificial scaffolds supporting the regeneration of damaged tissue must be non-cytotoxic [70]. Metabolic activity assay with MG-63 osteoblast-like cells was used to evaluate cytotoxicity effect of three-components biopolymeric scaffolds, cross-linked by EDC/NHS [44,52]. The results of the biological tests are presented in Figure 13. The metabolic activity of the cells was measured at three timepoints: 1, 4 and 7 cell culture days. The irreversible reaction of resazurin to resorufin, which takes place during resazurin test, is proportional to the aerobic respiration. That is why this test is frequently applied to assess mitochondrial metabolic activity [71]. Three types of materials were tested: Coll/CTS-based, SF/Coll-based and SF/CTS-based with 10, 20 and 30% addition of a third biopolymer (silk fibroin, chitosan and collagen, respectively), cross-linked by EDC/NHS. In the previous manuscript concerning the ternary-based mixtures, no studies were performed on mixtures without cross-linking, because they are unstable in the aqueous conditions [44,52]. All types of the scaffolds were cytocompatible with respect to MG-63 cells. Cell metabolic activity improved along with the time of culture for all the tested materials. As can be seen in Figure 13, after the first day of cell culture, metabolic activity stayed at similar levels for all the tested scaffolds. The biggest differences can be seen after 4 days of cell culture. Additionally, after 7 days, significant differences could be detected between the different types of materials. The group of scaffolds based on SF/Coll (50/50) mixture with addition of chitosan were characterized by the highest metabolic activity at both time points. This is a result very similar to that of materials based on the same biopolymers, with similar compositions, but cross-linked with a different agent—glyoxal [52]. Relating this with two-component materials based on the silk fibroin and chitosan blends, it can be seen that at 6 h after MG-63 cells seeding, they started to adhere to the walls of the scaffold material, and after 24 h, the majority of cells had completed adhesion and cell volumes increased, but a small number of cells showed growth projections [37]. Going further, on the third day of the experiment, the cells were tightly connected to each other, and the walls and pores of the scaffold material were completely covered with cells [37]. Parekh et al. [72] also prepared in vitro study on silk fibroin/chitosan with MG-63 cells and compared it with silk fibroin/collagen materials. It was found that the viability of the cells on the silk fibroin/collagen and silk fibroin/chitosan scaffold was comparable, and no significant differences between these two scaffolds could be observed, which is consistent with our research with three-component materials [72].

Materials containing a 50/50 mixture of silk fibroin and collagen with addition of chitosan ensure the best environment for the interaction with osteoblast-like cells of all the studied scaffolds. This conclusion also indicates that there is no toxic effect of the cross-linking agent on cell viability.

## 4. Conclusions

Biocompatible and cytocompatible porous scaffolds based on three-component mixtures of collagen, chitosan and silk fibroin were obtained. These scaffolds were successfully chemically cross-linked by EDC/NHS. It was found that those materials presented a high swelling degree (the most swollen material reached 7580 ± 50%) and moisture content ranging between 9.73 ± 1.08 and 18.26 ± 0.24 g per 100 g of dry sample. The mechanical parameters were dependent on the composition of the materials. None of the evaluated scaffolds were cytotoxic to osteoblast-like cells. These materials were biocompatible and created an adequate environment for cell viability. Three dimensional scaffolds based on 50/50 SF/Coll mixture with addition of chitosan give the most promising results and they can be a subject of future in vivo tests on animals. New biopolymeric composites based on collagen, chitosan and silk fibroin, cross-linked by EDC/NHS can be potentially used in biomedical applications, especially for biomaterials to be used in bone tissue regeneration.

## Data Availability

The data presented in this study are available on request from the corresponding author.
